# The subjective perception of the happiness of older adult residents in Colombia

**DOI:** 10.3389/fmed.2023.1055572

**Published:** 2023-05-05

**Authors:** Alejandra Segura, Doris Cardona, Angela Segura, Carlos Arturo Robledo, Diana Isabel Muñoz

**Affiliations:** CES University - Catholic University of Valencia San Vicente Martir, Medellín, Colombia

**Keywords:** older person, happiness, aging, subjective well-being, mental health

## Abstract

**Introduction:**

Happiness is understood as the perception of subjective well-being, it can be a quality, a result, or a state characterized by well-being or satisfaction that every person wants to achieve. In older adults, this satisfaction is a sum of lifelong achievements and triumphs; However, some factors influence this ideal.

**Objective:**

Analyze demographic, family, social, personal, and health factors associated with the subjective perception of happiness in older adults, using data from a study conducted in five cities in Colombia, in order to make a theoretical contribution in the search for improvement of their physical, mental and social health.

**Materials and methods:**

A quantitative, cross-sectional, analytical study was carried out, using primary source information, obtained with 2,506 surveys from voluntary participants aged 60 and over, who had no cognitive impairment, and who reside in urban areas but not in long-term centers. The variable happiness (classified as high or moderate/low) was used for: (1) A univariate explorative characterization of older adult, (2) a bivariate estimation of the relationships with the factors studied, and (3) a multivariate construction of profiles through multiple correspondences.

**Results:**

67.2% reported high happiness levels, with differences by city: Bucaramanga (81.6%), Pereira (74.7%), Santa Marta (67.4), Medellín (64%), and Pereira (48.7%). Happiness was explained by the absence of risk of depression and little hopelessness, strengthened psychological well-being, a perception of high quality of life, and living in a functional family.

**Conclusion:**

This study provided an overview of possible factors that can be enhanced and strengthened with public policies (structural determinant), community empowerment, family strengthening (intermediate determinant), and educational programs (proximal determinant). These aspects are included in the essential functions of public health, in favor of mental and social health in older adults.

## Introduction

The term happiness, sometimes interchangeable with life satisfaction, is used for the affective appraisal of life and is synonymous with the hedonic level of effect or subjective well-being in three components: affective well-being, eudemonic well-being, and evaluative well-being (1). Happiness is an essential factor for healthy aging, and there is a growing interest in the role that positive affect plays in improved health, lower mortality, decreased morbidity, and functional independence, in both community and clinical populations (2).

Happiness is understood as a vital experience of the human condition. In general, humans desire and seek happiness. It consists of the sensory, affective, and evaluative experiences of feeling good and how well life goes (3). Higher levels of well-being increase survival in older adults (4).

The importance of the concept of happiness is evident; The General Assembly of the United Nations declared March 20 as the “International Day of Happiness” to recognize it as a universal aspiration to be considered in government policies (5). According to the report on world happiness 2021, Colombia ranked 66/146 (6); it dropped in comparison to the previous years.

Latin Americans have a high subjective perception of happiness, as reported by its citizens (7). This is a paradox due to the region’s high poverty rates, inequities, and low access to social and health services, compared to some European countries (8). In different countries, such as Chile, Bhutan, France, and England, the measurement of happiness has been incorporated into their censuses and household surveys (9).

In a study carried out in three Colombian cities (2016), it was found that a better quality of life increases the probability that older persons are happier. The study found that the conditions related to education, health, habits, community, and family support contribute to this perception of happiness, with the coastal city of Barranquilla having the happiest group (10). Likewise, in the city of Manizales, it was found that the experience of well-being is strengthened by access to different material resources (11).

Affective, family and social bonds, and social support, generate positive feelings, this subjective expression of personal well-being also includes an evaluation of one’s own emotional state and satisfaction with life (12). The concept of happiness must be observed in its integrality. A study conducted in Bhutan suggests that such perception is holistic, that is, it is obtained when the spiritual, material, and social needs of the human being are achieved. This welfare state generates a vision of balanced progress, related to good health and the World Health Organization (WHO) considers it an indicator of development (13).

Physical, psychological, and social well-being are part of a good aging process, it includes individual, social, cultural, economic, and environmental aspects. High levels of subjective well-being, such as feelings of happiness, enjoyment, personal aspirations, and achievements can increase health, fitness, and longevity, leading to happy older people living longer and better lives (14).

The fundamental properties of happy behavior are personal satisfaction, possession of goods, achievement of goals, and the absence of negative emotional states (15). Despite efforts to quantify the state of happiness, this notion is still under construction and constantly re-evaluated as it has been studied with a multidisciplinary approach.

Happiness is an affective state of satisfaction subjectively experienced by an individual in possession of desired goods (16). It is from this definition that Alarcón (15) proposed that happiness has the following characteristics: (a) Happiness is an emotion of satisfaction experienced by an individual in his or her inner life, with subjective elements that allow for the individualization happiness; (b) Happiness is a state of conduct, which refers to its temporary nature, so it can be enduring or perishable; (c) Happiness implies the possession of goods, that is, the desired objects cause the individual to be happy; (d) The things that produce happiness have diverse traits (material, ethical, esthetic, psychological, religious, social, etc.) to which an individual assigns a certain value; (e) At a given time and in a particular cultural or society, group desires could coincide in the aspiration of a certain asset or specific assets. This definition includes the substantive characteristics of happiness.

In general, the measurement and study of happiness in older adults can be analyzed as proposed by Lyubomirsky (17), who considers that happy people are successful through multiple domains of life, for example, friendship, income, job performance, and health. Those who perceive themselves as happy adapt better to everyday experiences with decision-making, the perception and interpretation of social situations, and the recovery from negative events such as failure. Happiness in old age and its relationship with the improvement of physiological and immune parameters (18) have been shown in the results of medical treatments. These also improve the perception of levels of quality of health care (19).

## Materials and methods

### Objective

We sought to analyze the demographic, family, social, personal, and health factors associated with the subjective perception of happiness in older adults, in five Colombian cities (Bucaramanga, Medellín, Pereira, Popayan, and Santa Marta), to contribute to the improvement of their physical, mental, and social health.

### Design

A quantitative, cross-sectional, analytical, primary source study was carried out based on 2,506 surveys of people over the age of 60, residing in the urban area of the cities of Bucaramanga, Medellín, Pereira, Popayan, and Santa Marta, participation was voluntary, and required written consent. Those with cognitive impairment according to the Mini-Mental State Examination (MMSE) (20) were excluded. Older adults under the influence of psychoactive substances, those with hearing limitations, and those residing in long-term care centers were also excluded.

### Population

The reference population included 681,715 people aged 60 or over, residing in the urban areas of Bucaramanga (96,748), Medellín (407,879), Pereira (78,127), Popayan (42,710), and Santa Marta (56,251) according to the population projections of the Administrative Department of National Statistics (21). The selection of the cities corresponded to two criteria: geographical location, for which the country was divided into five regions, and a city was taken from each of these (north, south, eastern, western, and central). The second criterion was the size of the older adult population, seeking to have cities with different population sizes. In each city, approximately 500 older people were selected, corresponding to the sample size required to achieve the representation of the variables of interest. The selection was carried out by cluster sampling in two stages: (a) random selection of 51 neighborhoods in each city, and (b) random selection of two blocks per neighborhood (102 per city). The final unit of analysis was all older adults residing in houses in the selected blocks. Older adults were surveyed simultaneously in all five cities, by a group of trained surveyors in each city, at different times of day and different days of the week. The final probabilities of selection and expansion factors were calculated for each older adult in each of the cities studied. Finally, the sampling error was 1.7%.

### Description of variables

Independent variables were grouped into demographic, family, social, personal, and health factors. These variables were measured with validated scales, prior authorization of their authors, and signing of the written consent by participants. The ethical considerations were approved by an Institutional Ethics Committee and by the entities funding the study (titled *Salud y Bienestar mental de la persona mayor, en cinco ciudades de Colombia, año 2021*, in its original language).

### Happiness

The Reynaldo Alarcón Lima scale (15) has the purpose of measuring happiness in the subscales: absence of deep suffering, satisfaction with life, personal fulfillment, and joy of living. The scale is made up of 27 Likert-type items, with five alternatives: totally agree, agree, neither agree nor disagree, disagree, and totally disagree. It allows happiness to be classified into three categories: high, moderate, and low. Previous validations showed that it is a reliable scale, with an acceptable internal consistency, with a Cronbach’s alpha of 0.84 (22). The scale was adapted and validated for older adults with 14 items grouped into four dimensions (23) and applied to other population groups in Latin America: Venezuela (24), Argentina (25), and Mexico (26).

### Data analysis

To characterize adults according to demographic, family, social, personal, and health factors, qualitative variables were taken, and frequency measures were used. Descriptive measures were calculated for quantitative variables, and the Kolmogorov–Smirnov test was used to determine normality.

To estimate the subjective perception of happiness in older adults, hypothesis tests were used, such as Chi-square, with value of *p*, and a statistical association was considered with a value of *p* of < 0.05. The dependent variable was recategorized as high (totally agree + agree) and moderate-low (neither agree nor disagree + disagree + totally disagree), to build comparable groups.

For the purpose of associating happiness with demographic, family, social, personal, and health status factors, the Chi-square test was used. Crude measures of association (prevalence ratios—PRc) were calculated; and subsequently, the association was adjusted with those that were significant (*p* < 0.05), with potentially confounding variables following those described in the literature on the subject and that did not present collinearity, obtaining from this adjusted association measures (RPa) with their respective confidence intervals, through stratified analysis and logistic regression techniques for explanatory or adjustment purposes.

A multiple correspondence model was estimated to look at the conditions of older adults with high and those with a moderate/low perceptions of happiness. For the statistical analysis of the data, the statistical package IBM SPSS Statistics for Windows version 25 (IBM Corp., Armonk, NY, United States) was used.

This article is derived from a study entitled “Mental health and well-being of older adults, in five cities of Colombia, year 2020” (*Salud y Bienestar mental de la persona mayor, en cinco ciudades de Colombia, año 2021*, in its original language). It was approved by the ethics committee of the CES University, classified as having minimal risk (Act 134 of May 23, 2019) and participants were required to sign informed written consent.

## Results

### Characteristics of older adults according to demographic, family, social, personal, and health factors

Information was obtained from 2,506 older adults, residents of five cities in Colombia: Bucaramanga, Medellin, Pereira, Popayan, and Santa Marta, each representing approximately 20% of the total sample. In relation to sex, Bucaramanga had the highest rate of female participants with 62.6%, men ranged from 37.4 to 54.25% between the cities. It was found that five out of every ten older adults were between 60 and 69 years old. The mean age was approximately 68, with a range of 45 years, from 60 to 105, which was the oldest. The most frequent marital status reported was, no partner, representing more than 57.5% approximately, although it is noteworthy that for the city of Santa Marta three out of five older adults are married or in a free union. It was found that 3 out of 100 older adults were not health system affiliation (health insurance), with greater intensity in Santa Marta. Six out of ten stated that they have no income, and of those who reported income, 50% earn around COP 500.000, and 75% earn around COP 980.000 (Table 1).

**Table 1:** Percentage distribution of older adults, according to demographic, family, social, personal, and health status factors.

Characteristics	City										Total	
	Bucaramanga		Medellín		Pereira		Popayan		Santa Marta			
	*n*	%	*n*	%	*n*	%	*n*	%	*n*	%	*n*	%
**Sex**												
Male	187	37.4	199	39.8	266	52.7	230	45.9	271	54.2	1,153	46.0
Female	313	62.6	301	60.2	239	47.3	271	54.1	229	45.8	1,353	54.0
**Age group**												
60–69	278	55.6	328	65.6	285	56.4	223	44.5	295	59.0	1,409	56.2
70–79	173	34.6	137	27.4	156	30.9	191	38.1	166	33.2	823	32.8
80 or over	49	9.8	35	7.0	64	12.7	87	17.4	39	7.8	274	10.9
**Marital status**												
Single	91	18.2	110	22.0	166	32.9	132	26.3	104	20.8	603	24.1
Married—partners	195	39.0	215	43.0	181	35.8	202	40.3	272	54.4	1,065	42.5
Separated—divorced	69	13.8	80	16.0	79	15.6	50	10.0	34	6.8	312	12.5
Widowed	145	29.0	95	19.0	79	15.6	117	23.4	90	18.0	526	21.0
**Healthcare provider affiliation**												
Yes	489	97.8	491	98.2	486	96.2	495	98.8	480	96.0	2,441	97.4
No	11	2.2	9	1.8	19	3.8	6	1.2	20	4.0	65	2.6
**Income category**												
Less than 1 min. Salary	207	41.4	199	39.8	118	23.4	238	47.5	92	18.4	854	34.1
Between 1 and 2 min. Salaries	57	11.4	16	3.2	15	3.0	15	3.0	59	11.8	162	6.5
More than 2 min. Salaries	12	2.4	2	0.4	2	0.4	2	0.04	32	6.4	50	2.0
Without income	224	44.8	283	56.6	370	73.3	246	49.1	317	63.4	1,440	57.5
**Risk of depression**												
Normal	334	66.8	178	35.6	177	35.0	393	78.4	176	35.2	1,258	50.2
Clinical depression	166	33.2	322	64.4	328	65.0	108	21.6	324	64.8	1,248	49.8
**Hopelessness**												
Normal or asymptomatic	190	38.0	108	21.6	17	3.4	158	31.5	70	14.0	543	21.7
Light	246	49.2	284	56.8	262	51.9	261	52.1	397	79.4	1,450	57.9
Moderate–severe	64	12.8	108	21.6	226	44.8	82	16.4	33	6.6	513	20.5
**Resilience**												
Low	454	90.8	498	99.6	502	99.4	470	93.8	493	98.6	2,417	96.4
High	46	9.2	2	0.4	3	0.6	31	6.2	7	1.4	89	3.6
**Mistreatment**												
Not mistreated	174	34.8	100	20.0	232	45.9	306	61.1	232	46.4	1,044	41.7
Have suffered some kind of mistreatment	326	65.2	400	80.0	273	54.1	195	38.9	268	53.6	1,462	58.3
**Psychological wellbeing**												
Great strength	256	51.2	56	11.2	166	32.9	233	46.5	238	47.6	949	37.9
Without strength	244	48.8	444	88.8	339	67.1	268	53.5	262	52.4	1,557	62.1
**Quality of life**												
Low	124	24.8	203	40.6	231	45.7	104	20.8	102	20.4	764	30.5
Moderate	166	33.2	240	48.0	151	29.9	223	44.5	279	55.8	1,059	42.3
High	210	42.0	57	11.4	123	24.4	174	34.7	119	23.8	683	27.3
**Compulsive gambling**												
Without problems	51	54.8	20	42.6	20	35.1	10	30.3	5	11.6	106	38.8
Some problems	42	45.2	27	57.4	24	42.1	23	69.7	27	62.8	143	52.4
Probable compulsive gambler	0	0.0	0	0.0	13	22.8	0	0.0	11	25.6	24	8.8
**Family functionality**												
Normal	432	86.4	344	68.8	370	73.3	421	84.0	343	68.6	1910	76.2
Light dysfunction	37	7.4	90	18.0	98	19.4	52	10.4	106	21.2	383	15.3
Moderate dysfunction	13	2.6	21	4.2	20	4.0	20	4.0	27	5.4	101	4.0
Severe dysfunction	18	3.6	45	9.0	17	3.4	8	1.6	24	4.8	112	4.5
**Drug misuse**												
No consumption	428	85.6	364	72.8	410	81.2	458	91.4	411	82.2	2071	82.6
1 substance	55	11.0	106	21.2	55	10.9	36	7.2	71	14.2	323	12.9
2 or more substances	17	3.4	30	6.0	40	7.9	7	1.4	18	3.6	112	4.5
**Of incapacity**												
Without risk	30	6.0	8	1.6	4	0.8	15	3.0	13	2.6	70	2.8
High risk	470	94.0	492	98.4	501	99.2	486	97.0	487	97.4	2,436	97.2
**Happiness**												
High	408	81.6	320	64.0	246	48.7	374	74.7	337	67.4	1,685	67.2
Low to moderate	92	18.4	180	36.0	259	51.3	127	25.3	163	32.6	821	32.8
**Life satisfaction**												
High	447	89.4	412	82.4	323	64.0	398	79.4	456	91.2	2036	81.2
Low to moderate	53	10.6	88	17.6	182	36.0	103	20.6	44	8.8	470	18.8
**Personal fulfillment**												
High	430	86.0	406	81.2	347	68.7	367	73.3	421	84.2	1971	78.7
Low to moderate	70	14.0	94	18.8	158	31.3	134	26.7	79	15.8	535	21.3
**Positive attitude to life**												
High	235	47.0	140	28.0	160	31.7	280	55.9	108	21.6	923	36.8
Low to moderate	265	53.0	360	72.0	345	68.3	221	44.1	392	78.4	1,583	63.2
**Enjoyment of life**												
High	287	57.4	251	50.2	264	52.3	341	68.1	149	29.8	1,292	51.6
Low to moderate	213	42.6	249	49.8	241	47.7	160	31.9	351	70.2	1,214	48.4

Five Colombian cities included, 2021.

It was found that more than half of older adults may have an episode of clinical depression, and three out of five manifested mild hopelessness, although it is noteworthy that in the case of Santa Marta, this increases to approximately four. In relation to the ability to face new challenges or opportunities, they state that they have low resilience (Table 1).

### Happiness and its associated factors

When analyzing behavior each of the factors that could be related to happiness, statistical differences were found in income, risk of depression, hopelessness, psychological well-being, quality of life, and family function. These conditions being the ones used to adjust the measure of association (Table 2).

**Table 2:** Demographic, family, social, personal, and health factors are associated with the happiness of older people.

Characteristics	Happiness		*X* ^2^	*p*-value	PRc (CI 95%)
	High *n* (%)	Moderate to low *n* (%)			
**Sex**					
Male	764 (45.3)	389 (47.3)	0.925	0.336	1.00
Female	921 (54.6)	432 (52.6)			1.02 (0.97–1.08)
**Age group**					
60–69	939 (55.7)	470 (57.2)	0.676	0.713	1.00
70–79	557 (33.0)	266 (32.3)			1.01 (0.95–1.07)
80 or over	189 (11.2)	85 (10–3)			1.03 (0.94–1.12)
**Marital status**					
Single	401 (23.7)	202 (24.6)	0.613	0.893	1.00
Married—Partners	725 (43.0)	340 (41.4)			1.02 (0.95–1.09)
Separated—divorced	209 (12.4)	103 (12.5)			1.00 (0.91–1.10)
Widowed	350 (20.7)	176 (21.4)			1.00 (0.92–1.08)
**Healthcare provider affiliation**					
Yes	1,640 (97.3)	801 (97.5)	0.120	0.728	1.00
No	45 (2.67)	20 (2.43)			1.03 (0.87–1.21)
**Categorized income**					
Less than 1 min. Salary	594 (35.2)	260 (31.6)	23.893	**<**0.001	1.00
Between 1- and 2-min. Salaries	130 (7.71)	32 (3.89)			1.15 (1.05–1.26)
More than 2 min. Salaries	39 (2.31)	11 (1.33)			1.12 (0.96–1.30)
Without income	922 (54.7)	518 (63.0)			0.92 (0.86–0.97)
**Risk of depression**					
Clinical depression	668 (39.6)	580 (70.6)	212.226	**<**0.001	1.00
Normal	1,017 (60.3)	241 (29.3)			1.51 (1.42–1.60)
**Hopelessness**					
Moderate—severe	179 (10.6)	334 (40.6)	361.949	**<**0.001	1.00
Light	1,027 (60.9)	423 (51.5)			2.02 (1.79–2.29)
Normal range or asymptomatic	479 (28.4)	64 (7.79)			2.52 (2.23–2.85)
**Resilience**					
Low	1,624 (96.3)	793 (96.5)	0.071	0.790	1.00
High	61 (3.62)	28 (3.41)			1.02 (0.88–1.17)
**Mistreatment**					
Not mistreated	687 (40.7)	357 (43.4)	1.671	0.196	1.00
Have suffered some kind of mistreatment	998 (59.2)	464 (56.5)			1.03 (0.98–1.09)
**Psychological well-being**					
Without strength	919 (54.5)	638 (77.7)	125.957	**<**0.001	1.00
Great strength	766 (45.4)	183 (22.2)			1.36 (1.29–1.44)
**Quality of life**					
Low quality of life	346 (20.5)	418 (50.9)	290.970	**<**0.001	1.00
Moderate quality of life	746 (44.2)	313 (38.1)			1.55 (1.42–1.69)
High quality of life	593 (35.1)	90 (10.9)			1.91 (1.76–2.08)
**Compulsive gambling**					
No gambling problems	72 (37.3)	34 (42.5)	1.983	0.370	1.00
Some gambling problems	106 (54.9)	37 (46.2)			1.09 (0.92–1.28)
Probable compulsive gambler	15 (7.77)	9 (11.2)			0.92 (0.65–1.28)
**Family functionality**					
Severe dysfunction	37 (2.19)	75 (9.13)	206.655	**<**0.001	1.00
Moderate dysfunction	36 (2.13)	65 (7.91)			1.07 (0.74–1.56)
Slight dysfunction	189 (11.2)	194 (23.6)			1.49 (1.12–1.98)
Normal	1,423 (84.4)	487 (59.3)			2.25 (1.73–2.93)
**Drug misuse**					
No consumption	1,400 (83.0)	671 (81.7)	0.0.16	0.699	1.00
1 substance	212 (12.5)	111 (13.5)			0.97 (0.89–1.05)
2 or more substances	73 (4.33)	39 (4.75)			0.96 (0.83–1.10)
**Risk of incapacity**					
No risk	54 (3.20)	16 (1.94)	3.207	0.073	1.00
High risk	1,631 (96.7)	805 (98.0)			0.86 (0.76–0.98)

PRc: crude prevalence ratio; CI: confidence interval. Five Colombian cities included, 2021.

Likewise, it is possible that for every person who is at risk of clinical depression and is happy, 1.51 persons do not have any risk of depression and are happy, but when doing the multivariate analysis, with the other variables, it was found that those who did not present this risk are 1.16 higher than those with clinical depression. It is observed in the crude association measures of the five final variables, that the prevalence ratio is underestimated, and once possible confusion factors were controlled, the strength of the association increased, preserving the statistical association in the relationship. Similarly, there is an increase when there is a better quality of life PR 1.91 (1.76–2.08) and there is no hopelessness 2.52 (2.23–2.85; Table 3).

**Table 3:** Crude and adjusted measures of the happiness of older adults, according to demographic, family, social, personal, and health factors.

Characteristics	*X* ^2^	*p* Value	PRc (IC 95%)	*p*-value	PRa (CI 95%)
**Risk of depression**					
Clinical depression	212.226	**<**0.001	1.00		1.00
Normal			1.51 (1.42–1.60)	<0.001	2.16 (1.761–2.65)
**Hopelessness**					
Moderate to severe	361.949	**<**0.001	1.00		1.00
Light			2.02 (1.79–2.29)	<0.001	6.075 (4.39–8.58)
Normal range or asymptomatic			2.52 (2.23–2.85)	<0.001	3.25 (2.57–4.10)
**Psychological well-being**					
Without strength	125.957	**<**0.001	1.00		1.00
Great strength			1.36 (1.29–1.44)	0.003	1.40 (1.12–1.75)
**Quality of life**					
Low quality of life	290.970	**<**0.001	1.00		1.00
Medium quality of life			1.55 (1.42–1.69)	<0.001	1.84 (1.48–2.293)
High quality of life			1.91 (1.76–2.08)	<0.001	3.17 (2.35–4.28)
**Family functionality**					
Severe dysfunction	206.655	**<**0.001	1.00		1.00
Moderate dysfunction			1.07 (0.74–1.56)	<0.001	4.14 (2.61–6.55)
Slight dysfunction			1.49 (1.12–1.98)	<0.001	2.015 (1.22–3.312)
Normal			2.25 (1.73–2.93)	0.451	1.27 (0.679–2.38)

PRc: Crude prevalence ratio; PRa: Adjusted prevalence ratio; CI: Confidence interval. Five Colombian cities included, 2021.

### Profile of older adults according to their subjective perception of happiness

**Figure 1:**
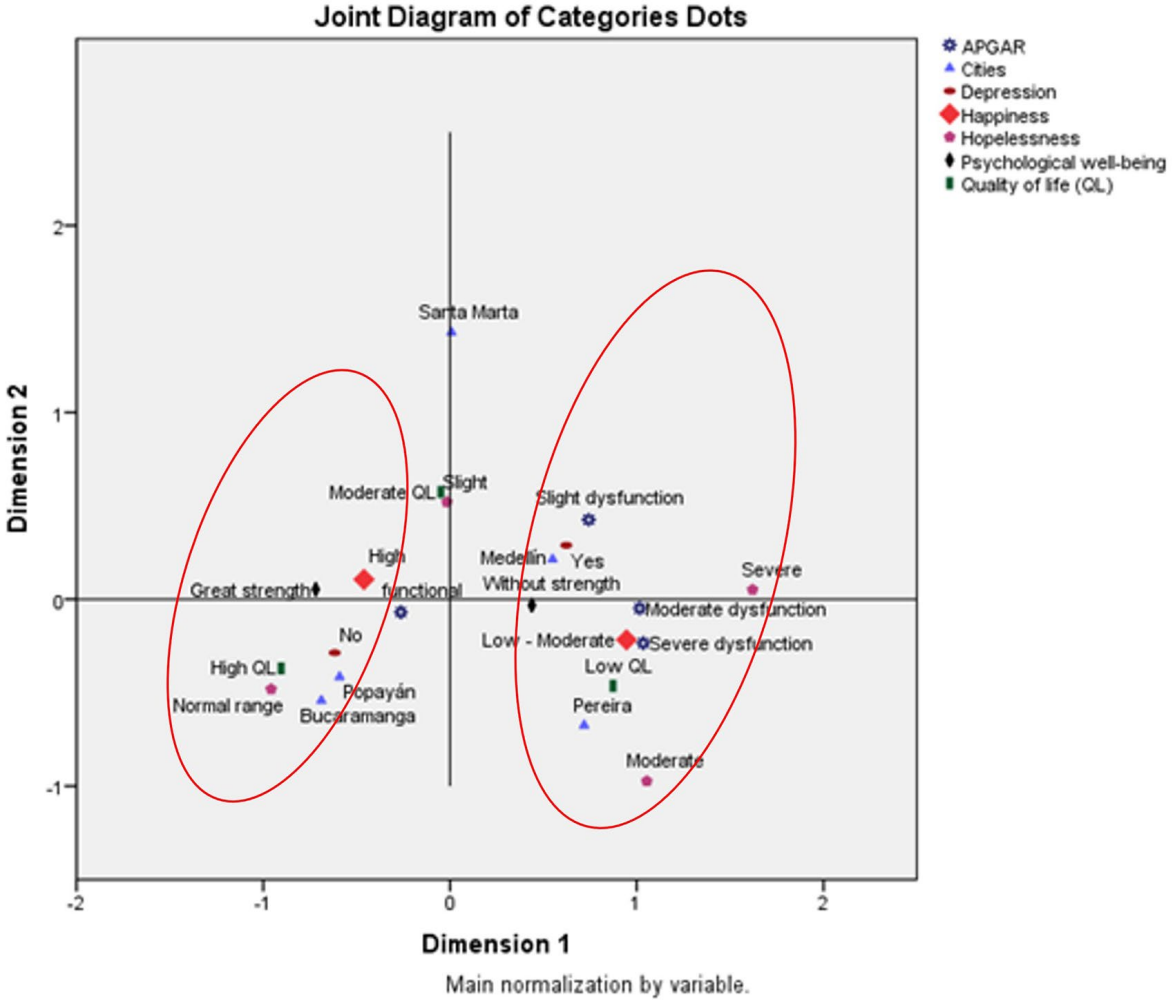
Profile of older adults, according to the subjective perception of their happiness. Five Colombian cities included, 2021 (Sans serif -SPSS).

A profile of people who are not happy, present clinical depression, have a low quality of life, present severe or moderate hopelessness, or have a dysfunctional family was identified. It presented with greater intensity in Medellín and Pereira. The profile of happy older adults who have family functionality and normal depression live in Bucaramanga, Popayan, and Santa Marta, have a moderate and high quality of life, and are very strong in terms of their psychological well-being, an important aspect to mention in this profile was the grouping that occurred in each of the categories of analysis (Figure 1).

## Discussion

Happiness is part of the component of subjective well-being that includes a broad category of phenomena among which are the emotional responses of people, the domains of satisfaction, and global judgment regarding life satisfaction (27). Given accelerated demographic changes and the challenges that they bring, happiness as a focus for the care of older adults should be considered by aging and old age policymakers, public health, social, and environmental science professionals, among others. It is not enough just to maintain health but to provide general well-being (28), and it is known that this is a key objective for society as a whole, since it leads to the happiness of its members (29).

In this study, two out of three older adults reported, according to the Lima Scale, a high happiness perception. A previous study carried out in 2016 found, with this same measurement, that 85.6% of older adults from three Colombian cities were in the happy or totally happy category (10). This shows a reduction in happy older adults, an aspect also seen at the population level that deserves attention because of aging populations. In the latest World Happiness Report (30), recently cited in a press release, Colombia is ranked 66th among the happiest countries in the world. The happiness index score in Finland, recognized as the happiest country in the world, with a score of 7,821, while Colombia scored 5,871 (30). Although the general happiness in the Colombian population has been declining in location (−3.84% variation) after being one of the happiest in the world, it now stands far from some South Americans (Uruguay has the best index of the region and is in position 30), happiness among older adults is higher than other countries. For example, in one part of Asia, it has been documented that the proportion of adults aged 60 years and over who are happy is around 55% (31). A recent population-based study in Iran reported that less than half of older adults reported an acceptable level of happiness (32). Likewise, studies in contexts similar to Colombia, such as Mexico, reported that, adjusting for age, the proportion of happy older adults is very small (33). However, Singapore, for example, has reported proportions of happiness in older adults as high as 96% (34).

Although these differences may have an important effect on the regional context in which people age, factors closer to the individual play a fundamental role. For older adults, happiness is related to subjective well-being, and, in this sense, it is shown as a complex construct, since it is conditioned by a variety of factors and determinants (some closer than others). Subjective well-being, life satisfaction, optimism, and positive emotions, all related to happiness, have been documented to improve health and longevity. Therefore, identifying the factors associated with happiness and the profile of older adults with a high subjective perception of happiness is important to guide strategies that provide a positive sense of life, especially during aging, both individual and collective, that translates into better indicators of happiness.

This study found that the happiness of older adults was explained mainly by the absence of the risk of depression, being asymptomatic to hopelessness, strengthening their psychological well-being, having a perception of high quality of life, and living in a functional family. Previous evidence has shown that several of these factors act jointly to determine the happiness of older adults, contribute to its mediation, and even intervene in it (35). In this sense, authors have proposed the concept of the multidimensionality of happiness, which is divided into four domains of life according to an extensive review of the literature: family, financial well-being, health and quality of life, and social interactions (36).

Among the factors that determine the happiness of older adults and that have been reported with greater consistency in studies are those that are related to social support: community and friends (37), living with a partner (38) and a functioning family (39). The happiness of older adults is more likely when they have a family, participate in social activities, and feel satisfied with their neighborhood environment (38) aspects that, in turn, explain, in part, the differences between older people with depression, hopelessness, and low perception of quality of life and psychological well-being.

These domains of support (social, friends, and family), manage to predict happiness, and this, in turn, is positively related to life satisfaction. One of the most recent studies reported by (37), managed to reveal significant differences in the happiness index among older adults who live alone (6.22 ± 2.11) compared to those who live with their partner (6.76 ± 1.99) and with their families (6.46 ± 1.94). Additionally, they found that older adults who live with their families showed greater happiness (OR = 0.80, 95% CI = 0.65–0.99) and that those who lived with their spouse increased their happiness when contact with friends was higher (OR = 0.69, 95% CI = 0.56–0.84) (39). For its part, a study by Saber et al. (32) proposed a relationship between happiness and social support, finding that the highest mean of this support was among those older adults who were happier and suggested, therefore, that it is urgent to address the social needs and communication networks of older people, as these can have a direct impact on their state of health and happiness. A study in three Colombian cities found that those with good functional families, even mild or moderate dysfunction, have a chance greater than 27% of being happy compared to those with severely dysfunctional families. It also found that the profile of happy older adults is given in part by having a functional family. Previous similar studies in our context have reported that two out of three older adults people have functional families (40) and, although this study did not find an association with the marital status of older adults, it has been indicated that this is an important characteristic that affects their well-being since greater satisfaction with life is found among married or accompanied older adults (39), which is also part not only of family formation but also, on occasions, an indicator of family functioning and perceived support of the couple.

Under this concept of the multidimensionality of happiness, and of conditions such as health and family, among other determinants, there are also associated factors that are related to each other and that end up defining the happiness and satisfaction of older adults (29, 31). Studies have shown that having close family ties protects against family dysfunction. Those who live in nursing homes far from their families and do not have social support, show more depressive symptoms, and their quality of life is lower (29, 41). The absence of depression, which this study found to be part of the happiness profile, has also been documented to have a mediating role in the relationship between happiness and living with a partner (38). A study that sought to determine the factors that influence the happiness of more than 14,000 older adults who live alone found that depression, as well as the quality of life, had a preponderant role in affecting the happiness of older adults (42). Most of the studies in this age group have specific approaches such as gender, people who live alone, on a low income, the institutionalized, and those with morbid conditions (for example, type 2 diabetes) among others. In general, the role played by depression as a factor influencing the happiness of older adults is consistently found. A recent systematic review (31) reported that the degree of depression had a strong impact on life satisfaction.

Quality of life and happiness largely depend on health status, and aging is a complex process with physical, psychological, and social changes, which can lead to illness and disability, and further reduce levels of happiness. An illness such as depression, for example, or a disability, can affect independence by limiting individual actions, and thus impairing quality of life, reducing the probability of being happy (35, 38). Authors such as Kim have suggested that family income, depression, subjective levels of stress, subjective levels of health, quality of life, and lack of medical services influence the happiness of older adults, mainly those who live alone (41).

In this sense, loneliness is a phenomenon of great concern due to its high incidence and impact. Hopelessness, specifically the loss of motivation and negative expectations about the future, are critical aspects of the development of feelings of loneliness in older adults (43). One of the factors in this study that characterized older adults who are not happy was hopelessness, and only one in five older adults do not suffer from it. This has been considered an important determinant of health and death, and, although it is a predictor of suicide (44) and loneliness (45), among other outcomes, it has become a modifiable risk factor for older adults, so this finding deserves attention and intervention (46). Additionally, evidence has also documented that hopelessness, in combination with depression and dissatisfaction with life, predicts conditions of psychological frailty in at least 68.4% of cases (46) and, although there is no specific evidence of hopelessness and its direct relationship with happiness in older adults, its mediating role in the relationship between psychological vulnerability and happiness in younger population groups has already been alerted (46, 47).

Although our study did not include an analysis of adjusted association measures in the final explanatory model, income was a condition associated with happiness (invariant analysis). More than half of the older adult population does not receive any income, and this reduces the probability of being happy by 8%. Previous evidence has shown that old age is linked to poverty and that the proportion of older people who do not receive any income exceeds 25% (48). Older people lack opportunities in the labor market, and the vast majority work in the informal sector (36). This implies that older people require economic support from relatives (36). Financial capacity is a determining factor for life satisfaction among older adults, as those with a better self-assessed economic capacity than others are generally more satisfied with their lives (31).

Happiness is a broad concept that includes several related elements such as life satisfaction, a good life, a better life, well-being, and quality of life (49). The profile of happy older adults, as well as the factors associated with happiness, are related to each other and, therefore, attending to them should involve an integrative approach that covers several fronts to achieve successful aging processes. Avoiding unhappiness through interventions that address issues related to social isolation, disability, and depression is recommended (34). Given the fact that the factors that explain it are modifiable, it is desirable that older adults be happy through the diverse options that, holistically, include individual conditions from psychological and physical well-being as well as family aspects, and social and community support.

A limitation of this study was the fact that other conditions such as those of a functional type such as physical activity, which is related as one of the determinants of this state among older adults were not explored. Previous evidence has reported that regular physical activity is an essential factor that influences happiness, even slight happiness, as it provides relaxation (50) and, in this sense, its study also allows its implementation as a prevention and care strategy. There might be another limitation in addressing happiness as the same concept during the various stages of aging and some characteristics of older adults. It has been reported, for example, that the perception of happiness changes with age because priorities and what makes people happy change. Blanchflower states that the reasons for relative happiness in old age are many, anchored by lower levels of stress and responsibility, and encouraged by free time (51). They are also conditioned by gender, marital status, economic conditions, social support, and functional families (52) as well as by the happiness motivation that derives from those hedonic or eudemonic motives. Although the association of some of these conditions was explained and how they constitute the profile of the happy older adult, it is recommended that future studies address these differences through stratified analysis, or by estimating the mediating role in the various relationships that lead to happiness.

## Conclusion

Happiness has been of interest in various parts of the world to different cultures and populations, and globally with the world report. Specifically, this research provides a theoretical contribution to the factors that can make old age a satisfying, calm, and gratifying part of life, as current evidence has shown that health problems and social situations take on a more important role at older ages. In this way, the first great conclusion is that happiness is not simply the result of depressive states, illness, loneliness, hopelessness, low quality of life, and not having social support, among others, but that it is related to a variety of characteristics, conditions, and processes, which accumulate throughout life.

It has also been identified that happiness has broad ramifications in the course of life and is affected, conditioned, and determined by personal, social, and economic relationships, biological risk factors, health behaviors and healthy habits, leisure, and use of time, as well as the health situation. This calls on institutions and actors to implement spaces in which these aspects are strengthened much more, with the care that happiness is modifiable when it improves the health and well-being of older adults or even with mood swings.

This study provided a systematic overview of the possible factors that can be enhanced and strengthened with public policies (structural determinant), community empowerment and family strengthening (intermediate determinant), and educational programs (proximal determinant), aspects included in the essential functions of public health, in favor of the mental and social health of older adults.

## Data availability statement

The original contributions presented in the study are included in the article/supplementary material, further inquiries can be directed to the corresponding authors.

## Ethics statement

The studies involving human participants were reviewed and approved by Comité Institucional de Ética en Investigación en Humanos, Universidad CES. The patients/participants provided their written informed consent to participate in this study.

## Author contributions

AS, DC, CR, and AS: material preparation, data collection and analysis, and writing—original draft preparation. AS and DC: conceptualization. AS: methodology. AS, DC, CR, DM, and AS: formal analysis and investigation. DM: writing—review and editing. DC: funding acquisition. Resources: Minciencias. All authors contributed to the article and approved the submitted version.

## Funding

This work was financially supported by MinCiencias (Ministry of Science, Technology, and Innovation of Colombia; Code 122884467945, Contract No. 425-2020, Grant 844 de 2019) and the CES University.

## Conflict of interest

The authors declare that the research was conducted in the absence of any commercial or financial relationships that could be construed as a potential conflict of interest.

## Publisher’s note

All claims expressed in this article are solely those of the authors and do not necessarily represent those of their affiliated organizations, or those of the publisher, the editors and the reviewers. Any product that may be evaluated in this article, or claim that may be made by its manufacturer, is not guaranteed or endorsed by the publisher.
